# A comparison of principal component regression and genomic REML for genomic prediction across populations

**DOI:** 10.1186/s12711-014-0060-x

**Published:** 2014-11-05

**Authors:** Christos Dadousis, Roel F Veerkamp, Bjørg Heringstad, Marcin Pszczola, Mario PL Calus

**Affiliations:** Animal Breeding and Genomics Centre, Wageningen University, PO Box 338, Wageningen, 6700, AH The Netherlands; Department of Animal and Aquacultural Sciences, Norwegian University of Life Sciences, PO Box 5003, Ås, N-1432 Norway; Animal Breeding and Genomics Centre, Wageningen UR Livestock Research, PO Box 338, Wageningen, 6700, AH The Netherlands; GENO Breeding and A. I. Association, PO Box 5003, Ås, N-1432 Norway; Department of Genetics and Animal Breeding, Poznan University of Life Sciences, Wolynska 33, Poznan, 60-637 Poland

## Abstract

**Background:**

Genomic prediction faces two main statistical problems: multicollinearity and *n* ≪ *p* (many fewer observations than predictor variables). Principal component (PC) analysis is a multivariate statistical method that is often used to address these problems. The objective of this study was to compare the performance of PC regression (PCR) for genomic prediction with that of a commonly used REML model with a genomic relationship matrix (GREML) and to investigate the full potential of PCR for genomic prediction.

**Methods:**

The PCR model used either a common or a semi-supervised approach, where PC were selected based either on their eigenvalues (i.e. proportion of variance explained by SNP (single nucleotide polymorphism) genotypes) or on their association with phenotypic variance in the reference population (i.e. the regression sum of squares contribution). Cross-validation within the reference population was used to select the optimum PCR model that minimizes mean squared error. Pre-corrected average daily milk, fat and protein yields of 1609 first lactation Holstein heifers, from Ireland, UK, the Netherlands and Sweden, which were genotyped with 50 k SNPs, were analysed. Each testing subset included animals from only one country, or from only one selection line for the UK.

**Results:**

In general, accuracies of GREML and PCR were similar but GREML slightly outperformed PCR. Inclusion of genotyping information of validation animals into model training (semi-supervised PCR), did not result in more accurate genomic predictions. The highest achievable PCR accuracies were obtained across a wide range of numbers of PC fitted in the regression (from one to more than 1000), across test populations and traits. Using cross-validation within the reference population to derive the number of PC, yielded substantially lower accuracies than the highest achievable accuracies obtained across all possible numbers of PC.

**Conclusions:**

On average, PCR performed only slightly less well than GREML. When the optimal number of PC was determined based on realized accuracy in the testing population, PCR showed a higher potential in terms of achievable accuracy that was not capitalized when PC selection was based on cross-validation. A standard approach for selecting the optimal set of PC in PCR remains a challenge.

**Electronic supplementary material:**

The online version of this article (doi:10.1186/s12711-014-0060-x) contains supplementary material, which is available to authorized users.

## Background

For many years, dairy cattle breeding programs have been very successful in identifying the best animals via progeny-testing schemes. Progeny-testing was first implemented in Denmark and was soon used all over the world [1]. One drawback of the progeny-testing scheme in dairy cattle breeding is the long generation intervals, which limits the response to selection, despite the high accuracy of selection achieved.

In order to reduce the generation interval by trying to obtain more accurate estimated breeding values (EBV) before progeny information is available, the use of molecular markers in connection with phenotypes to predict genetic merit has been investigated for some time [2]. Recent advances in molecular techniques have made large-scale applications of such techniques possible. In 2001, Meuwissen et al. [3] showed by simulation that genome-wide dense markers can adequately be used to estimate breeding values with a considerably high accuracy. Prediction of these EBV based on marker information is known as genomic prediction, and the subsequent selection step is known as genomic selection (GS). In GS, DNA information is used to predict the genetic merit of young animals, in order to reduce generation intervals. In recent years, GS has been implemented in dairy cattle breeding programs [4–8] and has been described as the most promising molecular application in livestock [9].

In practise, genomic prediction involves two steps. First, the effect of each SNP (single nucleotide polymorphism) is estimated in a reference population that consists of animals with both known phenotypes and marker genotypes. In the second step, genomic breeding values (GEBV) of young animals are estimated using only their marker information, to rank the animals for selection.

Despite the fact that several methods have been presented to estimate SNP effects, there are still many important questions and problems to be addressed, including statistical issues. These statistical issues concern mainly multicollinearity in the SNP dataset, due to linkage disequilibrium (LD) among markers, which leads to unstable estimates in least-squares regression. Moreover, a major problem in the statistical models used to estimate SNP effects is that the number of variables that needs to be estimated (*p*) is much larger than the number of observations (*n*), thereby removing least squares from possible analysis methods. In the field of statistics, these problems are frequently overcome by using principal component analysis (PCA) and subsequent regression on the principle components (PC) (PCR; principal component regression) instead of on the original variables.

In general, PCA can be used to solve multicollinearity problems among predictor variables and to reduce the dimensional space. In genetic studies, PCA has been used mainly for population studies and has been a powerful tool to identify population structures and migration patterns, and to correct for stratification in association studies by capturing genetic variation [10–15]. One of the first applications of PCA in population genetics was by Menozzi et al. [16] to produce maps of human genetic variation across mainland regions.

Likewise, in animal breeding, PCA has recently been used to infer population clusters from different breeds [17] and to represent genotypes in the prediction of GEBV [18–21]. Daetwyler et al. [22] used PCA to investigate the impact of population structure on the accuracy of GEBV in a multi-breed sheep population. Results of these studies, which used either simulations or real data, describe PCA as a promising method for animal breeding to produce accurate GEBV. In these studies, the main benefits of using PCA were a significant reduction in data quantity (>90%) and fast computation. However, to date, there is only a limited number of studies based on real data that compare PCR for genomic prediction with a more commonly used genomic prediction model such as GBLUP (best linear unbiased prediction, in which the pedigree additive relationship matrix is replaced with a marker-derived relationship matrix) [23]. Since PCA is able to recover population structure, it may be expected that using this information is beneficial for genomic prediction applied to data with strong population structure. One such application is across-population genomic prediction, e.g. genomic prediction based on reference data that only includes data from other populations and not from the predicted population itself. Whether the ability of PCA to detect population structure is also beneficial in applications of across-population genomic prediction is currently unknown.

The main objective of this research was to investigate the potential of PCR for across-population genomic prediction, as applied to yield traits in Holstein cows from different countries. More precisely, the objectives were (i) to compare the predictive accuracy of PCR with a REML model that uses a genomic relationship matrix (GREML) and (ii) to investigate the effect of alternative methods of extracting and selecting PC on the accuracy of genomic predictions.

## Methods

### Data

We used 66 116 daily records up to 45 weeks in lactation for milk, fat and protein yields from 1609 first lactation Holstein heifers. Heifers originated from four countries, Ireland (IRL; Teagasc, Moorepark Dairy Production), United Kingdom (UK; Scottish Agricultural College), the Netherlands (NLD; Wageningen UR Livestock Research) and Sweden (SWE; Swedish University of Agricultural Science). The UK data included animals from two divergent selection lines, a line selected for high fat and protein yield and a control line that represents the UK national average for fat and protein yield [24]. These two lines were therefore considered as two groups (UK_1 and UK_2). All phenotypes were pre-adjusted to account for the mean overall lactation curve, herd, diet group, milking frequency, year-month of milk test-day by management group, and experimental treatments. For a full description, see [24,25]. For each animal, a single pre-adjusted phenotype was obtained as the average daily milk, fat and protein yields for lactation weeks 3 to 15, derived from individually predicted lactation curves. Descriptive statistics of the pre-adjusted phenotypes are in Table 1.

**Table 1:** **Descriptive statistics of pre-adjusted average daily data of the milk yield traits**

**Trait**	**Mean**	**SD**	**SE**	**Min**	**Max**	**n**
Milk yield (kg)	23.84	4.44	0.111	0.99	38.98	1609
Fat yield (kg)	0.93	0.18	0.004	0.12	1.79	1609
Protein yield (kg)	0.72	0.13	0.003	0.04	1.34	1609

All animals were genotyped within the RobustMilk project (www.robustmilk.eu) with the Illumina BovineSNP50 Beadchip (Illumina Inc., San Diego, CA) containing 54 001 SNPs. Quality control checks on the SNPs used the following criteria: (1) a GenCall score greater than 0.20 and a GenTrain score greater than 0.55 for individual genotypes; (2) a call rate greater than 95%; (3) a minor allele frequency greater than 0.01 in each country; and (4) no extreme deviation from Hardy Weinberg Equilibrium (*χ*^2^ < 600). After editing, 37 069 SNPs remained across the 29 autosomes and the X-chromosome.

### Reference and test datasets

The across-country dataset was split into five subsets and, in each analysis, four subsets were used as the reference set and the other one for testing. The first three test datasets included animals from only one country (Ireland, the Netherlands or Sweden), while the last two each contained one of the UK selection lines, such that each animal had its genomic breeding value predicted once for each trait and model. The number of cows in each subset ranged from 181 to 618 (Table 2). Accuracies of predicted genomic breeding values were calculated as Pearson correlations between the predicted genomic breeding values and the adjusted phenotypes within each test dataset (i.e. within country, and within line for the UK animals).

**Table 2:** **Number of cows with phenotype records and genotypes from Ireland, the Netherlands, Sweden and two divergent selection lines from UK**

**Population**	**Number of animals**
UK_1	206
UK_2	210
Sweden	181
Ireland	394
The Netherlands	618
**Total**	**1609**

### Principal component analysis

Assume a matrix **X** of order *(n* ***×*** *p)* where *n* individuals have been genotyped for *p* SNPs. The elements of this matrix may be 0, 1 or 2, representing the genotype of each individual for each SNP (0 and 2 for homozygotes and 1 for heterozygotes). The main idea of PCA is to reveal hidden structure in the data, to reduce the number of variables in the dataset, and to solve the multicollinearity problem (high correlation between columns in **X**). It extracts the most important information, in terms of variation, and re-expresses the original dataset in a simplified way. Thus, PCA aims at finding a small set *k (k < p)* of PC that explain as much of the variability in **X** as possible. This is achieved through an orthogonal transformation of the original dataset such that as much of the original variability as possible is included in the first few PC. So, PC are linear combinations of a set of random variables in **X**, i.e. the matrix **T** with PC is obtained by:

$$ \mathbf{T}={\mathbf{e}}^{\mathbf{\prime}}\mathbf{X}, $$

where **e** represents the eigenvectors derived from spectral decomposition of the covariance (or correlation) matrix of **X**. In genomic data, the covariance (correlation matrix) of the SNP genotypes (of order *p × p*) can be used or alternatively the similarity matrix of the individuals (**G** matrix, of order *n × n*). The first PC is then defined as the vector:

$$ {\mathbf{T}}_1={\mathbf{e}}_1^{\mathbf{\prime}}\mathbf{X}={e}_{11}{\mathbf{X}}_1+{e}_{12}{\mathbf{X}}_2+\cdots +{e}_{1p}{\mathbf{X}}_p, $$

which captures the maximum variance in **X**, with the constraint that **e**′**e** = 1. For all PC combinations, it holds that: *cov*(**T**_i_, **T**_j_ = 0) for all *i ≠ j (i,j = 1,2,…,p).*

The basis of PCA is either the spectral decomposition of the covariance (correlation) matrix of **X** or the singular value decomposition (SVD) of **X**. The SVD represents a more general view of the eigenvalue decomposition for non-square matrices **X**. In general, PCA based on SVD and eigen decomposition are expected to yield similar results if **X** is square and symmetric [26]. Moreover, SVD on an *n* ***×*** *p* matrix **X** is expected to yield the same results as on its *p* ***×*** *p* correlation matrix.

### Principal component regression and genomic prediction

The concept of PCR, i.e. the use of PC in regression has been around for quite some time in the field of statistics [27,28]. For application in genomic prediction, first consider the general model to predict breeding values based on marker genotypes:

$$ \mathbf{y}=\mathbf{1}\mu +\mathbf{X}\mathbf{b}+\mathbf{e}, $$

where values in **e** are iid $\sim N\left(\mathbf{0},\mathbf{I}{\upsigma}_{\mathrm{e}}^2\right),$**y** is a vector of phenotypic records, **1** is a vector of ones, *μ* is the overall mean, **X** is a matrix (centred and possibly scaled) containing SNP genotypes, **b** is a vector of additive effects of all SNPs, **e** is a vector of residual effects, **I** is the identity matrix and ${\upsigma}_{\mathrm{e}}^2$ is the residual variance. The initial step for a PCR model is to perform PCA on the genotype matrix **X***(n × p).* For this purpose, we used SVD via the function “prcomp” in R [29], which works as follows. Consider the SVD of **X**, **X** = **UΣV**′, where **U** and **V** are the left and right singular vectors of **X**, **V**′ is the transpose of **V** and **Σ** is a diagonal matrix containing the singular values. The matrix **T** (*n × k*) of PC scores is then calculated as:

$$ \mathbf{T}=\mathbf{X}\mathbf{V}=\mathbf{U}\Sigma {\mathbf{V}}^{\mathbf{\prime}}\mathbf{V}=\mathbf{U}\Sigma, $$

where *k < r*, where *r* is the rank of **X**, and **V***(p × k)* is the loading matrix derived from the SVD of **X**, which defines weights to the original **X** variables in each PC.

### PCA based on the reference dataset

Principal component analysis was initially performed only on the SNP matrix of the reference dataset, where the **T** matrix of PC was calculated as **T**_r_ **= X**_r_**V**, where r denotes the reference dataset. The **V** matrix that was extracted from the reference dataset was also used to construct the **T** matrix for the test dataset as **T**_t_ **= X**_t_**V**, where **X**_t_ contains the genotypes of the test dataset.

Following from the above, the PCR model that was applied is:

$$ \mathbf{y}=\mathbf{1}\mu +\mathbf{Tg}+\mathbf{e}, $$

where **T** is the matrix of PC, and **g** is a vector with regression coefficients for each PC in **T**. In this case, the derived transformed SNP effects (i.e. the values in **g)** are treated as fixed effects in contrast to what is commonly used in genomic prediction models that perform simultaneous regression on SNP genotypes treating SNP effects as random [30].

### PCA based on all animals

In a second approach, PCA was performed on the matrix with all SNP genotypes, where genotypes of all reference and test datasets were included. For application in the PCR model, the **T** matrix was split in parts relating to the reference and test datasets (**T**_**r**_ and **T**_**t**_, respectively), using methods that are briefly described in Additional file 1: Figure S1. In this approach, hereinafter referred to as semi-supervised PCR (SSPCR), the genotypic information of the individuals to be predicted is partly included in the training dataset of the prediction model. This is because the axes of variation, i.e. the singular vectors and the singular values of the SVD were extracted using all genomic information available in the dataset. This concept of semi-supervised PCA was borrowed from computer science and face recognition analysis [31,32].

### Selection of PC for inclusion in the PCR models

Two methods were tested to select sets of PC to be used in the subsequent PCR models. In the first method, PC were ranked based on decreasing eigenvalues (variation in the explanatory variables, i.e. the genotypes), which will be referred to as PCR_eigen. In the second method, the PC were ranked based on their contribution to the sum of squares (ss) of the regression (variation in the response variable), referred to as PCR_ss. These contributions were obtained from a PCR model for which only phenotypes and genotypes of the animals of the reference dataset were included.

### Selection of the optimal model in PCR

Once the order in which the PC should be added to the model is established, the question is how many PC should be used in the subsequent PCR model used for genomic prediction. There is no general consensus on which strategy should be followed for this. Inspecting plots of eigenvalues (via the so-called “scree plots” that plot the PC ranked based on decreasing eigenvalues) or keeping the number of PC that capture a given percentage of the original variation are two among a variety of methods (see [33] for a detailed review). In our analyses, a cross-validation (CV) approach within the reference dataset (as in [33]) was chosen in order to obtain the “optimum” number of PC to include in the PCR, which will be further used in the section on prediction of the test dataset. For CV, the reference dataset was either split by country (and line in the case of UK), which will be referred to as stratified CV hereafter, or split randomly in a 5-fold CV. In both these CV approaches, all PC were added in the PCR model one by one and the minimum mean squared error (MSE) of the predictions within the reference dataset was used as the target function to be optimized, which is briefly described in Additional file 2: Figure S2. Both CV approaches were performed using the R package “plsdof” [34], with the appropriate modification for the semi-supervised PCA.

### GREML model

For the GREML model, the following individual animal model with a genomic relationship matrix was fitted in ASReml-R [35]:

$$ \mathbf{y}=\mathbf{1}\mu +\mathbf{W}\mathbf{u}+\mathbf{e}, $$

where **u** is a vector of additive genetic effects for any of the considered traits and **W** is the design matrix that links **u** to the phenotypic records in **y**. For additive genetic and residual effects, the following normal distributions were assumed: $\mathbf{u}\sim N\left(\mathbf{0},\mathbf{G}{\upsigma}_{\mathrm{u}}^2\right)$ and $\mathbf{e}\sim N\left(\mathbf{0},\mathbf{I}{\upsigma}_{\mathrm{e}}^2\right).$ Note that this is a genomic BLUP model but with estimation of variances ${\upsigma}_{\mathrm{u}}^2$ and ${\upsigma}_{\mathrm{e}}^2$ together with estimation of the breeding values using restricted maximum likelihood (REML). Therefore, this model is more appropriately referred to as genomic REML (GREML) [36]. The genomic relationship matrix was calculated following VanRaden [23] as:

$$ {\mathbf{G}}_{\mathbf{VR}}=\frac{\mathbf{Z}{\mathbf{Z}}^{\mathbf{\hbox{'}}}}{2{\displaystyle \sum {p}_i\left(1\hbox{-} {p}_i\right)}}, $$

where *p*_*i*_ is the frequency at SNP *i* for which the homozygous genotype is coded 2, calculated across all genotyped animals, and **Z** is derived from genotypes of animals in the reference dataset by subtracting 2*p*_*i*_ from a matrix **X** that specifies the marker genotypes for each individual as 0, 1 or 2. Following Yang et al. [37], **G**_**VR**_ was regressed back towards **A** (the pedigree relationship matrix) to account for errors in the estimation of **G**_**VR**_, resulting in the computation of the genomic relationship matrix **G** as:

$$ \mathbf{G}=b\times {\mathbf{G}}_{\mathbf{VR}}+\left(1-b\right)\times \mathbf{A}, $$

where *b* is estimated according to Yang et al. [37]. The value of $\widehat{b}$ ranged from 0.975 to 0.997 across different bins of pedigree-based relationships. So, although in theory this adjustment of **G**_**VR**_ improves the properties of the **G** matrix [37], in our case the adjusted **G** matrix was very close to the original matrix and is therefore expected to yield very similar predictions.

## Results

### Characterization of the data

**Figure 1:**
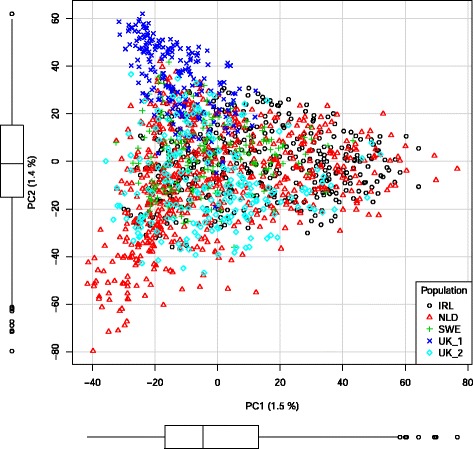
**Scatterplot of the first two principal components (PC1 vs. PC2).** Principal component analysis performed on the whole dataset, with data from Ireland (IRL), the Netherlands (NLD), Sweden (SWE) and two divergent selection lines from United Kingdom (UK_1 and UK_2).

PCA was performed on all SNP genotypes to investigate differences in genotypes between the Holstein populations included in this study. Based on the plot of the 1st against the 2nd PC (Figure 1), one of the selection lines of the UK population could be distinguished from the rest with the first PC. However, it should be noted that the 1st and 2nd PC captured only 1.5% and 1.4% of the total original variability of the SNP data, respectively (Table 3). Comparison of relationships based either on pedigree or genomic relationships also confirmed that the UK_1 population had the weakest average relationship with the other populations (Table 4). In nearly all cases, standard deviations of the genomic relationships were higher than those of pedigree-based relationships, which indicates that the use of SNP information explains more variation in relationships than pedigree information. Averages and standard deviations of relationships were always higher within populations than between populations. This confirms that relationships among the five populations were low and that genomic predictions in these data indeed were “across populations”, in the sense that the reference data always included data only from other populations and not from the predicted population itself.

**Table 3:** **Cumulative proportion of the original variability captured by principal components (PC)**

**Number of PC**	**Cumulative proportion (%)**
1	1.5
2	2.9
37	20
138	40
326	60
668	80
967	90

Principal component analysis was performed on the whole dataset.

**Table 4:** **Average (Av) and SD of pedigree and genomic relationships within and between countries (and selection lines)**

			**UK_1**	**UK_2**	**Sweden**	**Ireland**	**NL**
Av	Within^1^	Pedigree	0.0612	0.0763	0.0534	0.0556	0.0792
		Genomic	0.0560	0.0226	0.0268	0.0182	0.0096
	Between^1^	Pedigree	0.0295	0.0490	0.0385	0.0424	0.0499
		Genomic	−0.0088	−0.0026	−0.0024	−0.0038	−0.0028
SD	Within	Pedigree	0.0894	0.0819	0.0919	0.0767	0.0645
		Genomic	0.0958	0.0860	0.0953	0.0788	0.0654
	Between	Pedigree	0.0220	0.0322	0.0284	0.0305	0.0332
		Genomic	0.0265	0.0331	0.0283	0.0311	0.0342

^1^Averages (Av) and SD are computed for relationships within each population (Within) and for relationships between each population and all other populations (Between).

### GREML versus optimal PCR and SSPCR

Accuracies of genomic predictions obtained using GREML, PCR and SSPCR models, after determining the optimal number of PC based on the reference data, are in Table 5. On average, across test datasets and traits, GREML outperformed the PCR and SSPCR models. Across the test datasets, the performance of the PCR and SSPCR models was closest to that of GREML for milk with PCR_eigen and 5-fold CV (0.15 vs. 0.14), and for fat with SSPCR_ss and 5-fold CV (0.07 for both models). For protein, the maximum accuracy obtained with PCR and SSPCR was 0.02 achieved in four cases (PCR_eigen and 5-fold CV, PCR_ss and stratified CV, SSPCR_ss and 5-fold CV, and SSPCR_ss and stratified CV) versus 0.05 for GREML.

**Table 5:** **Accuracies** ^**1**^ **obtained for the PCR** ^**2**^ **and GREML** ^**3**^ **models**

**Test**	**Trait**	**GREML**	**PCR_eigen**		**PCR_ss**		**SSPCR_eigen**		**SSPCR_ss**	
			**5-fold**	**stratified**	**5-fold**	**stratified**	**5-fold**	**stratified**	**5-fold**	**stratified**
UK_1	Milk	0.26*	0.21 (249)	0.16 (204)	0.17 (142)	NA^4^ (0)	0.10 (71)	NA (0)	0.13 (821)	0.22 (537)
	Fat	0.07	−0.08 (35)	−0.01 (103)	0.02 (95)	0.11 (1)	−0.03 (40)	NA (0)	0.07 (859)	*0.12 (614)*
	Protein	−0.01	−0.05 (121)	−0.09 (69)	−0.02 (91)	NA (0)	−0.13 (91)	NA (0)	0.03 (910)	*0.05 (570)*
UK_2	Milk	0.10	0.12 (233)	0.12 (217)	0.05 (342)	*0.14 (13)*	0.12 (89)	0.13 (10)	0.04 (941)	0.03 (527)
	Fat	−0.02	−0.13 (96)	−0.12 (37)	−0.11 (35)	−0.09 (28)	−0.08 (33)	−0.08 (27)	0.03 (909)	*0.05 (523)*
	Protein	0.01	−0.03 (89)	−0.06 (46)	−0.11 (91)	*0.06 (7)*	0.04 (83)	0.02 (10)	−0.06 (780)	−0.01 (505)
SWE	Milk	*0.16*	0.15 (181)	0.14 (187)	0.12 (184)	0.04 (19)	0.03 (64)	0.04 (15)	0.09 (895)	0.07 (529)
	Fat	0.09	0.10 (162)	0.10 (146)	0.04 (101)	0.07 (713)	0.07 (57)	−0.02 (1)	*0.16 (859)*	0.04 (680)
	Protein	0.06	0.00 (92)	−0.04 (48)	0.01 (82)	−0.06 (29)	−0.10 (17)	−0.10 (16)	*0.10 (1025)*	*0.10 (574)*
IRL	Milk	0.06	0.05 (277)	−0.10 (48)	−0.03 (207)	−0.08 (37)	−0.04 (35)	−0.13 (8)	*0.14 (717)*	0.13 (389)
	Fat	*0.08*	0.06 (121)	0.04 (51)	0.02 (100)	*0.08 (52)*	0.07 (37)	0.02 (14)	0.07 (712)	0.06 (315)
	Protein	0.04	0.05 (127)	0.04 (145)	0.00 (35)	−0.04 (173)	−0.05 (37)	−0.12 (12)	*0.12 (759)*	0.11 (421)
NLD	Milk	0.16	0.18 (50)	*0.19 (28)*	0.09 (55)	0.10 (7)	0.18 (25)	0.13 (2)	0.11 (95)	0.11 (95)
	Fat	*0.15*	0.11 (47)	0.10 (99)	0.05 (75)	0.06 (7)	0.07 (39)	0.07 (7)	0.10 (197)	0.11 (196)
	Protein	0.13	0.14 (31)	0.13 (34)	0.07 (42)	0.13 (7)	0.12 (28)	*0.16 (2)*	0.11 (48)	0.11 (48)
Average	Milk	*0.15*	0.14 (198)	0.10 (137)	0.08 (186)	0.05 (15)	0.08 (57)	0.04 (7)	0.09 (694)	0.02 (415)
	Fat	*0.07*	0.01 (92)	0.02 (87)	0.00 (81)	0.05 (160)	0.02 (41)	0.00 (10)	*0.07 (707)*	0.04 (466)
	Protein	*0.05*	0.02 (92)	0.00 (68)	−0.01 (68)	0.02 (43)	−0.02 (51)	−0.01 (8)	0.02 (704)	0.02 (424)
SD	Milk	0.08	0.06 (90)	0.12 (91)	0.08 (105)	0.10 (14)	0.08 (26)	0.12 (6)	0.04 (345)	0.07 (189)
	Fat	0.06	0.11 (53)	0.09 (44)	0.07 (28)	0.08 (310)	0.07 (9)	0.06 (11)	0.05 (295)	0.04 (204)
	Protein	0.05	0.08 (38)	0.09 (45)	0.07 (27)	0.09 (73)	0.10 (34)	0.13 (7)	0.08 (382)	0.05 (219)

For the PCR models, the PC included (numbers are presented in brackets) were selected based on cross-validation in the reference population data, either in a 5-fold random or stratified split to select the optimum PCR model in respect to minimum mean squared error. Analyses were performed for three traits and five test populations.

^1^Accuracies were calculated as Pearson correlations between the predicted genomic breeding values and the adjusted phenotypes; ^2^selection of PCs was based either on the eigenvalues (eigen) or the regression sum of squares (ss); two different methods of applying principal component analysis, either separately for reference and test parts (PCR) or on the whole dataset (SSPCR), were compared; ^3^a REML based model with a genomic relationship matrix; ^4^all animals received the same prediction; *in italics the highest accuracies for each population and trait.

### Comparison between optimal PCR and SSPCR

Accuracies of genomic prediction differed between the PCR and SSPCR models and also based on the two CV approaches that were used to obtain the optimum PCR (or SSPCR) model (Table 5). Interestingly, in some cases the stratified CV resulted in a null model, i.e. a model where only the intercept was included. In such cases, all predicted individuals had the same GEBV which was the mean of the reference dataset. This occurred only when predicting UK_1 and was independent of the method used (PCR or SSPCR), the approach of sorting the PC (eigen vs. ss), and trait. A closer look on the number of PC used in the various PCR and SSPCR models and in the CV methods (Table 5) showed that, in general, a stratified approach reduced the number of PC but also resulted in lower accuracy, on average, compared to using 5-fold random CV. Moreover, for all except the UK_1 fat predictions, SSPCR_eigen used fewer PC than PCR_eigen. In contrast, quite a large number of PC was included in the SSPCR_ss models for all traits and both CV approaches.

### GREML versus “best case scenario” of PCR and SSPCR

In the present study, a CV approach was used to select the PCR (or SSPCR) model that was used for genomic prediction. An additional objective was to investigate the full potential of PCR (or SSPCR) and the ability of CV to achieve this. To investigate this, the pattern of the accuracies when adding PC one by one in the model, was evaluated to identify the model with the highest accuracy (“best case scenario”). It should be noted that this is not possible in practical genomic prediction applications, because it involves the use of phenotypic information of the test dataset. Those best case scenarios PCR (or SSPCR) models always outperformed GREML (Table 6) and the optimal PCR and SSPCR models based on cross-validation (Table 5).

**Table 6:** **Highest accuracies** ^**1**^ **obtained for PCR models** ^**2**^ **versus those obtained with the GREML model** ^**3**^

**Test**	**Trait**	**GREML**	**PCR_eigen**	**PCR_ss**	**SSPCR_eigen**	**SSPCR_ss**
UK_1	Milk	0.26	0.44 (14)	0.36 (2)	0.45 (6)	0.23 (458)
	Fat	0.07	0.16 (776)	0.13 (3)	0.18 (1)	0.21 (219)
	Protein	−0.01	0.25 (14)	0.16 (1)	0.28 (7)	0.09 (1)
UK_2	Milk	0.10	0.16 (3)	0.15 (11)	0.19 (144)	0.15 (46)
	Fat	−0.02	0.08 (1061)	0.07 (751)	0.12 (593)	0.09 (1370)
	Protein	0.01	0.07 (1)	0.08 (2)	0.10 (151)	0.08 (233)
SWE	Milk	0.16	0.18 (1112)	0.16 (1060)	0.21 (365)	0.17 (344)
	Fat	0.09	0.22 (46)	0.10 (991)	0.16 (1425)	0.18 (871)
	Protein	0.06	0.11 (265)	0.08 (790)	0.25 (1424)	0.20 (1419)
IRL	Milk	0.06	0.15 (967)	0.12 (758)	0.14 (92)	0.19 (288)
	Fat	0.08	0.12 (954)	0.14 (572)	0.16 (790)	0.12 (965)
	Protein	0.04	0.12 (749)	0.09 (245)	0.16 (94)	0.13 (273)
NLD	Milk	0.16	0.21 (20)	0.17 (4)	0.19 (24)	0.17 (16)
	Fat	0.15	0.17 (794)	0.19 (400)	0.17 (585)	0.18 (78)
	Protein	0.13	0.17 (7)	0.16 (8)	0.17 (5)	0.17 (4)
Average	Milk	0.15	0.23 (423)	0.19 (367)	0.24 (126)	0.18 (230)
	Fat	0.07	0.15 (726)	0.13 (543)	0.16 (679)	0.16 (701)
	Protein	0.05	0.14 (207)	0.11 (209)	0.19 (336)	0.13 (386)
SD	Milk	0.08	0.12 (565)	0.10 (506)	0.12 (144)	0.03 (192)
	Fat	0.06	0.05 (398)	0.05 (373)	0.02 (511)	0.05 (540)
	Protein	0.05	0.07 (323)	0.04 (341)	0.07 (611)	0.05 (591)

Analyses were performed for three traits and five test populations.

^1^Accuracies were calculated as Pearson correlations between predicted genomic breeding values and adjusted phenotypes; ^2^selection of PC was based either on the eigenvalues (eigen) or the regression sum of squares (ss); two different methods of applying principal component analysis, either separately for reference and test parts (PCR) or on the whole dataset (SSPCR), were compared; ^3^a REML based model with a genomic relationship matrix.

### PCR in the “best case scenario”

**Figure 2:**
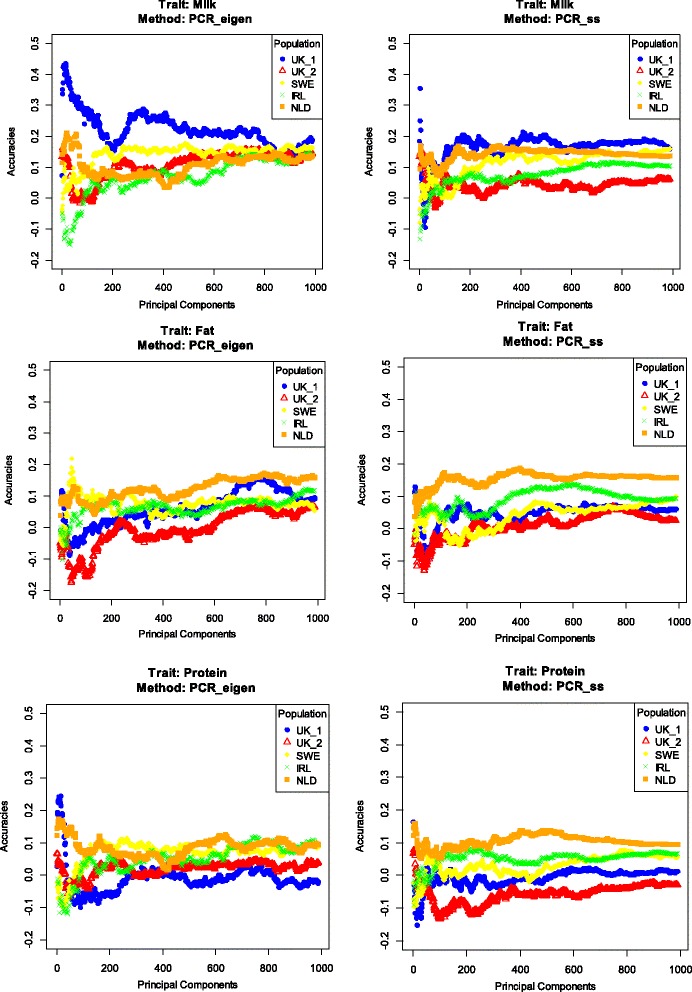
**Pattern of accuracies for principal component regression models with increasing numbers of principal components (PC).** PCA was performed in the reference dataset. Selection of PC was based on eigenvalues (left panel) or on the sum of square contributions (right panel). Traits analysed were average daily milk, fat and protein yields for test populations from Ireland (IRL), the Netherlands (NLD), Sweden (SWE) and two divergent selection lines from United Kingdom (UK_1 and UK_2).

For the common PCR case, where PCA was applied on the genotypic data from the reference dataset, the pattern of accuracies was evaluated for an increasing number of PC that were included in the model based on decreasing eigenvalues (Figure 2). Several interesting observations can be made from these results. The PCR_eigen method generally resulted in higher accuracies than the PCR_ss method (Figure 2; Table 6). Accuracies using PCR_ss were slightly higher in only three cases. The pattern of the accuracies, when an increasing number of PC was included in the models, differed between traits and populations. In many cases, using very few PC (usually less than 50) gave accuracies very close to the maximum obtained across the whole range of number of PC included (Figure 2).

### SSPCR in the “best case scenario”

**Figure 3:**
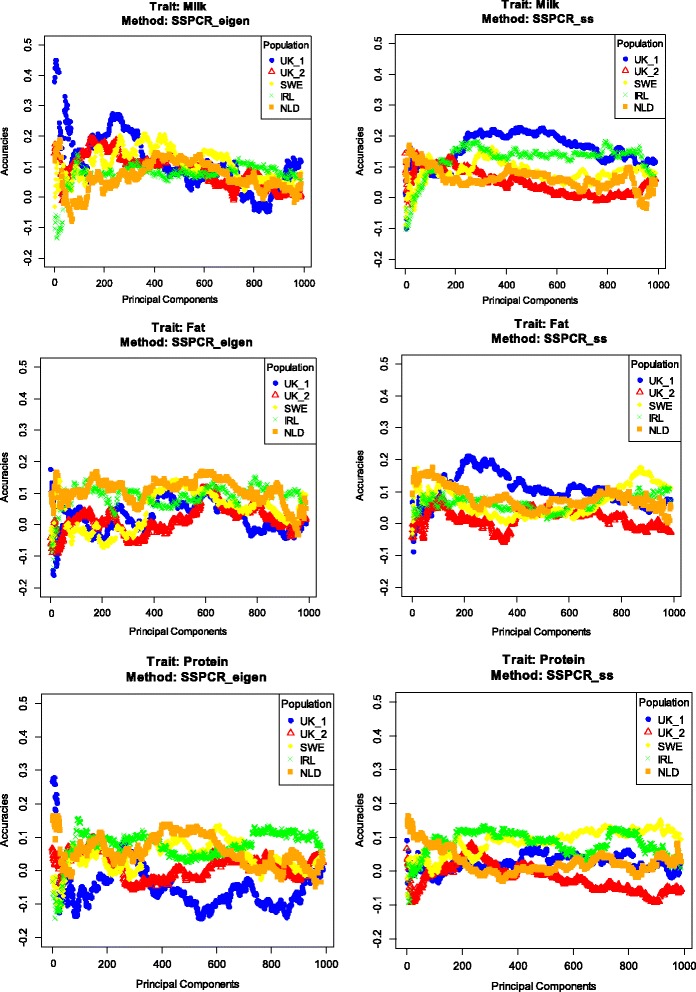
**Pattern of accuracies for semi-supervised principal component regression models with increasing numbers of principal components (PC).** PCA was performed on the whole dataset. Selection of PC was based on eigenvalues (left panel) or on the sum of square contributions (right panel). Traits analysed were average daily milk, fat and protein yields for test populations from Ireland (IRL), the Netherlands (NLD), Sweden (SWE) and two divergent selection lines from United Kingdom (UK_1 and UK_2).

For this model, the whole SNP dataset, i.e. both the reference and testing data, was included in the PCA, while only phenotypes of the reference subset were used to estimate the regression coefficients in PCR. In this approach, genomic information on the test dataset, such as LD, is incorporated in the weights on the SNPs applied in each eigenvector. In some cases, this SSPCR_eigen method resulted in a slight increase in accuracies and in a reduction in the number of PC needed to achieve the highest accuracies compared to PCR_eigen (Table 6, Figure 3). Interestingly, for the UK_1 population, accuracies of 0.45, 0.18 and 0.28 for milk, fat and protein yields were obtained with only the first six, one and seven PC, respectively. On average, SSPCR resulted in slightly higher accuracies than PCR when the genotypes of the test dataset were excluded from the PCA step.

## Discussion

Principal component regression enables data reduction in the regression model and solves problems of dependencies among variables (multicollinearity). The main advantage of PCR derives from the ability of PCA to capture a large proportion of the original variability of the dataset (e.g. >90%) in a small set of uncorrelated PC. As a result, generally a limited number of PC can replace the original variables with little loss of information. Based on this, we tested whether PCA and its regression (PCR) can provide a useful alternative method for genomic prediction. Our results showed that, on average, PCR yielded lower accuracies than the more commonly used GREML model, although it has the potential to yield considerably higher prediction accuracies than the GREML model. It should be noted that this potential was realized in the “best case scenario” that used both genotypic and phenotypic information from the test dataset animals to derive the optimal number of PC included in the model, which is not possible in practice. Nevertheless, the results of this scenario can be regarded as an upper limit of the achievable prediction accuracy, provided that the most appropriate PC can be selected in a practical application. Optimization (i.e. selection of PC) using the reference data by two different CV approaches (stratified vs. 5-fold random), proved to be unable to capitalize on the full potential of PCR, but still achieved levels of prediction accuracy close to those obtained with GREML. Although prediction accuracies appeared to be quite low for all models, it should be noted that the reported accuracies are Pearson correlations between observed phenotypes and predicted GEBV. Transforming those values to the accuracy of GEBV, which is defined as the correlation between true and predicted GEBV, involves division by the square root of the heritability of the trait. Since, for instance, the heritabilities of the adjusted phenotypes for milk yield used in our study ranged from 0.13 to 0.59 (results not shown) across countries, accuracies of GEBV for milk yield are predicted to be 1.3 to 2.8 times higher than the reported correlations.

Genomic relationships between reference and test datasets have been shown to have an important effect on prediction accuracy [38,39]. The average squared relationship between reference and test datasets has been shown to be a better predictor of accuracy of genomic prediction than the average relationship between reference and test datasets [38]. Since the variance of relationships is closely related to the average squared relationship, we compared the standard deviation of relationships and the average relationships between populations (Table 4). The on average low relationships between the populations in our data and, in particular, the lower variance of relationships between populations compared to within populations, predicted that accuracies of genomic predictions would be low, which was indeed the case. Although we focussed on commonly measured milk yield traits, our results can be extended to other traits such as feed intake, for which, pooling existing research herd data is the only option to enable genomic prediction [40]. In fact, pooling of such data has become possible by using genotypes, because models based on genomic relationships can overcome issues caused by low connectedness based on pedigree [41].

### Optimization of PCR and SSPCR models through cross-validation

An important question is how to select the optimal set of PC for the PCR model, using information from the reference data, such that the accuracy achieved is at least similar to the accuracy achieved with GREML. We used CV on the reference data to optimize the order and number of included PC. As a first observation, the “best” PCR model, i.e. the model obtained in the “best case scenario”, was never proposed with the CV approach. In our analyses, optimization of the CV was based on minimising the MSE. However, since the accuracy of EBV is important for animal breeding and affects response to selection, an alternative scenario could be to select the “best” PCR model after CV in the reference data based on maximum accuracy instead of minimum MSE.

The main advantage of PCR was that it reduced the dimension of the data by at least 96%. Despite the generally high reduction in data dimension, the highest accuracies after CV were achieved for a wide range of numbers of PC included in the PCR model, from only one to more than 1000 (Tables 5 and 6). This is a wider range than that reported in previous studies based on simulated data, in which the highest accuracies were achieved when the number of PC ranged from 250 to 350 [19,20]. However, it should be noted that for PCR_eigen, which is the most commonly used approach in the literature, the number of PC in the model was between 28 and 249 after CV and between 1 and 1112 for the “best case scenario”. By adding PC one by one in the model, it was shown that most PC affected the accuracy of predictions either positively or negatively and thus the trajectory of the accuracies was not stable but fluctuated (Figures 2 and 3). Moreover, in some cases the first few PC resulted in the highest accuracies. As a result, using empirical thresholds to select PC for inclusion in the model (e.g. PC that together explain more than 90% of the original variability in the SNP genotypes based on eigenvalues) simply does not result in the highest accuracies that can be achieved in PCR. Thus, the number and selection of PC for inclusion in the PCR model should be derived empirically for each dataset.

The semi-supervised PCR model also used genotypic information from the test dataset. Despite our expectation that this would improve accuracy of predictions, because the model would be forced to define PC that explain variation in the genotypes of the test dataset, this model on average performed less well than the PCR model (Table 5). However, for the best case scenario, when accuracies were evaluated across different numbers of PC based on the phenotypes of the test dataset, the semi-supervised approach did yield slightly higher accuracies than the PCR model (Table 6). This indicates that the semi-supervised PCR has the potential to perform at least equally well as PCR, although it appears that identifying the optimal set of PC for the semi-supervised PCR model is even more difficult than for the PCR model. In the context of genomic prediction, the semi-supervised PCR may be more relevant when large differences exist between the genotypes of the reference and the test datasets. The most obvious application is across-breed or -line genomic prediction, where one breed or line is used to predict another. In that case, the semi-supervised PCR model may be able to better target the variance of the predicted line or breed. It should be noted that including animals of the predicted breed in the reference data, e.g. [42,43], may yield a similar result, regardless of the model used.

### Investigating the importance of the principal components in regression

In other studies that used PCA for genomic prediction [18–21], the PC used only accounted for the variability captured in the original matrix **X** (SNPs) and not for the proportion of explained phenotypic variance in the reference population (as used here with PCR_ss in the common and semi-supervised approaches). However, it has been shown in statistical literature that the first principal components (accounting for most variation in **X**) can totally fail as predictors in PCR (in terms of accounting for variation in the response variable) and that even components that explain little variance in **X** can be important for prediction [28,44–46]. For instance, using Hald’s data, Hadi and Ling [47] showed that while the first three (out of four) PC accounted for 99.96% of the variability in **X**, they contributed nothing (zero sum of squares) to the fit of the regression model; instead, the last PC alone contributed everything. Thus, these authors proposed that the selection of PC should be based not only on the variance decomposition of the co-variables but also on the contribution of each PC to the regression sum of squares. However, despite the expectation that PCR_ss would yield at least equally accurate estimates as PCR_eigen, we observed the opposite in our analyses. It should be noted that PCR_ss selected PC based on associations with phenotype in the reference data. In that regard, PCR_ss is very similar to partial least squares regression [21,48]. Thus, our results suggest that the PC that show the strongest associations with phenotypes in the reference data do not necessarily have the strongest associations with the phenotypes in the testing data.

### GREML versus “best case scenario” for PCR and SSPCR

By investigating the pattern of the accuracies of the PCR models (Figures 2 and 3), we observed that some specific combinations of PC resulted in relatively high prediction accuracies, considering the limited size of the reference dataset, and also compared to GREML. In our analyses, the highest accuracies from the PCR models across the numbers of PC included outperformed those from the GREML models in all cases. The data reduction achieved by PCR solves the “small *n* large *p*” issue and thereby enables the use of fixed regression, as done in our study, rather than the random regressions commonly used in genomic prediction models. An important question is why PCR_eigen and SSPCR_eigen in the “best case scenario” achieved a higher accuracy than GREML, while it uses a linear transformation of the SNP data used in GREML. The most likely explanation is the fact that by using fixed regression, the model is able to put as much or as little emphasis on any PC, without shrinking the effects, following the associations with phenotypic data. Other genomic prediction models such as GREML assume equal contributions of each SNP to the total variability, and generally include shrinkage of effects of individual SNP effects by modelling them as random effects. Although the variance explained by SNPs in a shrinkage model can still depart substantially from the prior assumptions based on evidence from the data, especially if the reference population is large [30], estimated effects will still be affected by those prior assumptions. In this respect, PCR can be regarded as a variable selection method, albeit at the level of PC rather than individual SNPs. This implies that the accuracies reported for the “best case scenarios” provide an upper limit for the accuracy that could be achieved with variable selection models applied to PC rather than to SNPs.

### Further improvement of PCR

One of the underlying assumptions of PCA is linearity, such that the feature space is a linear transformation of the original data. In order to overcome the problem of linearity, Schölkopf et al. [49] considered nonlinear component analysis as a “kernel eigenvalue problem” and introduced the term “kernel PCA”. The use of kernels has already been introduced in genomic prediction models [50]. In addition, since Bayesian models are often used in genomic prediction, the use of probabilistic PCA [51], where maximum likelihood is used to extract PC, could also be proposed for future research in genomic prediction.

Concerning the selection of SNPs the target function in our study was to minimize the prediction MSE. As suggested above, an alternative could be to select PC in the CV procedure based on maximum prediction accuracy. In addition, more sophisticated techniques such as the combination of statistical methods like PCA, neural networks and genetic algorithms could be applied, as has already been tested in other fields [52]. However, a balance between benefits (e.g. higher prediction accuracies) and costs (e.g. computation time) should be taken into account.

### PCA in genetic studies

In general, PCA and multivariate analysis techniques have proven to be useful tools to extract information from markers. In addition, as an exploratory method, analyses with PCA can be performed without strong assumptions on the data (e.g. Hardy-Weinberg equilibrium, LD) [53]. A disadvantage of PCA is that it does not take the response variable into account. However, in our study, this did not affect accuracies negatively (comparing PCR_eigen and PCR_ss in the common and semi-supervised approach). Nevertheless, it remains necessary to be very careful when applying multivariate analysis to genomic data, especially when interpreting the results. Jombart et al. [53] provided a nice overview of a multivariate analysis application with genetic data and examined the incorrect use of multivariate analysis in different genetic datasets, as well as fallacies when interpreting the results. For instance, one assumption of PCA is that PC with large eigenvalues represent structure in the data, while those with low eigenvalues capture noise. This might not always be true and PC with small eigenvalues could contain predictive information, or perhaps reflect genotypes at a single SNP. Thus, PC should not be excluded from the analysis on the basis of their small contribution to the total variance in SNP genotypes. Our results confirmed that the optimum number of PC to be included in the PCR model can vary considerably across datasets and traits.

## Conclusions

Our results show that PCR results in genomic prediction accuracies that are generally slightly lower than those obtained with a GREML model. In general, selecting PC based on their eigenvalues resulted in higher accuracies than selecting PC on decreasing correlations to the response variable in the reference dataset. Inclusion of genotypic information of the test animals when extracting the PC, i.e. the semi-supervised approach, unexpectedly decreased the accuracy when PC were selected based on the reference dataset after cross-validation. The semi-supervised approach did, however, slightly increase the potential of the model, i.e. the highest accuracies that can be achieved, provided that it is possible to select the optimal set of included PC. While the pattern of prediction accuracies across included PC showed that PCR had a higher potential than GREML, the model that was selected by CV within the reference data could not capitalize on this potential. On average, 5-fold random CV for PCR outperformed stratified CV. However, to capitalize on the full potential of PCR in practical applications, it is still unclear what the best way to select PC to be included in the model is.

## Additional files

Additional file 1: Figure S1. Schematic overview of computation of PC in PCR and SSPCR models. PC were computed using either only genotypes of the reference data (PCR) or using genotypes of both the reference and test dataset (SSPCR). Additional file 2: Figure S2. Schematic overview of stratified and 5-fold cross-validation (CV) approaches, used to select PC in the model based on minimum mean squared error of the predictions within the reference dataset. The CV was performed by splitting the reference dataset by country (stratified CV), or by splitting it randomly in a 5-fold CV.

## References

[1] Johansson I (1960). Progeny testing methods in Europe. J Dairy Sci.

[2] Neimann-Sorensen A, Robertson A (1961). The association between blood groups and several production characteristics in three Danish cattle breeds. Acta Agric Scand.

[3] Meuwissen THE, Hayes BJ, Goddard ME (2001). Prediction of total genetic value using genome-wide dense marker maps. Genetics.

[4] Berry DP, Kearney F, Harris BL (2009). Genomic selection in Ireland. Interbull Bull.

[5] De Roos APW, Schrooten C, Mullaart E, Van der Beek S, De Jong G, Voskamp W (2009). Genomic selection at CRV. Interbull Bull.

[6] Ducrocq V, Fritz S, Guillaume F, Boichard D (2009). French report on the use of genomic evaluation. Interbull Bull.

[7] Wiggans GR, Sonstegard TS, VanRaden PM, Matukumalli LK, Schnabel RD, Taylor JF, Chesnais JP, Schenkel FS, Van Tassel CP, Sattler JD (2008). Genomic Evaluations in the United States and Canada: A Collaboration. Proceedings of International Commitee of Animal Recording; 16–20 June; Niagara Falls.

[8] Loberg A, Dürr JW (2009). Interbull survey on the use of genomic information. Interbull Bull.

[9] Sellner EM, Kim JW, McClure MC, Taylor KH, Schnabel RD, Taylor JF (2007). Board-invited review: applications of genomic information in livestock. J Anim Sci.

[10] McVean G (2009). A genealogical interpretation of principal components analysis. PLoS Genet.

[11] Novembre J, Stephens M (2008). Interpreting principal component analyses of spatial population genetic variation. Nat Genet.

[12] Paschou P, Drineas P, Lewis J, Nievergelt CM, Nickerson DA, Smith JD, Ridker PM, Chasman DI, Krauss RM, Ziv E (2008). Tracing sub-structure in the European American population with PCA-informative markers. PLoS Genet.

[13] Patterson N, Price AL, Reich D (2006). Population structure and eigenanalysis. PLoS Genet.

[14] Price AL, Patterson NJ, Plenge RM, Weinblatt ME, Shadick NA, Reich D (2006). Principal components analysis corrects for stratification in genome-wide association studies. Nat Genet.

[15] Reich D, Price AL, Patterson N (2008). Principal component analysis of genetic data. Nat Genet.

[16] Menozzi P, Piazza A, Cavalli-Sforza L (1978). Synthetic maps of human gene frequencies in Europeans. Science.

[17] Lewis J, Abas Z, Dadousis C, Lykidis D, Paschou P, Drineas P (2011). Tracing cattle breeds with principal components analysis ancestry informative SNPs. PLoS ONE.

[18] Dimauro C, Cellesi M, Pintus MA, Macciotta NPP (2011). The impact of the rank of marker variance–covariance matrix in principal component evaluation for genomic selection applications. J Anim Breed Genet.

[19] Macciotta NPP, Gaspa G, Steri R, Nicolazzi EL, Dimauro C, Pieramati C, Cappio-Borlino A (2010). Using eigenvalues as variance priors in the prediction of genomic breeding values by principal component analysis. J Dairy Sci.

[20] Pintus MA, Gaspa G, Nicolazzi EL, Vicario D, Rossoni A, Ajmone-Marsan P, Nardone A, Dimauro C, Macciotta NPP (2012). Prediction of genomic breeding values for dairy traits in Italian Brown and Simmental bulls using a principal component approach. J Dairy Sci.

[21] Solberg TR, Sonesson AK, Woolliams JA, Meuwissen THE (2009). Reducing dimensionality for prediction of genome-wide breeding values. Genet Sel Evol.

[22] Daetwyler HD, Kemper KE, van der Werf JHJ, Hayes BJ (2012). Components of the accuracy of genomic prediction in a multi-breed sheep population. J Anim Sci.

[23] VanRaden PM (2008). Efficient methods to compute genomic predictions. J Dairy Sci.

[24] Banos G, Coffey MP (2010). Short communication: Characterization of the genome-wide linkage disequilibrium in 2 divergent selection lines of dairy cows. J Dairy Sci.

[25] Veerkamp RF, Coffey MP, Berry DP, de Haas Y, Strandberg E, Bovenhuis H, Calus MPL, Wall E (2012). Genome-wide associations for feed utilisation complex in primiparous Holstein–Friesian dairy cows from experimental research herds in four European countries. Animal.

[26] Diamantaras KI, Kung SY (1996). Principal Component Neural Networks: Theory and Applications.

[27] Hotelling H (1957). The relations of the newer multivariate statistical methods to factor analysis. Br J Stat Psych.

[28] Jeffers JNR (1967). Two case studies in the application of principal component analysis. J R Stat Soc Series C (Appl Stat).

[29] R Development Core Team: *R: A Language And Environment For Statistical Computing*; [http://www.R-project.org/]

[30] de los Campos G, Hickey JM, Pong-Wong R, Daetwyler HD, Calus MPL (2013). Whole-genome regression and prediction methods applied to plant and animal breeding. Genetics.

[31] Roli F, Marcialis GL, Yeung DKJ, Fred A, Roli F, Ridder D (2006). Semi-Supervised PCA-Based Face Recognition Using Self-Training. Structural, Syntactic, and Statistical Pattern Recognition.

[32] Yu S, Yu K, Tresp V, Kriegel H-P, Wu M (2006). Supervised Probabilistic Principal Component Analysis. Proceedings of the 12th ACM SIGKDD International Conference on Knowledge Discovery and Data Mining; Philadelphia.

[33] Jolliffe IT (2002). Principal Component Analysis.

[34] Krämer N, Sugiyama M (2011). The degrees of freedom of partial least squares regression. J Am Stat Assoc.

[35] Butler D, Cullis B, Gilmour A, Gogel D (2009). ASReml-R reference Manual Release 3.0.

[36] Calus MPL, de Haas Y, Veerkamp RF (2013). Combining cow and bull reference populations to increase accuracy of genomic prediction and genome-wide association studies. J Dairy Sci.

[37] Yang J, Benyamin B, McEvoy BP, Gordon S, Henders AK, Nyholt DR, Madden PA, Heath AC, Martin NG, Montgomery GW, Goddard ME, Visscher PM (2010). Common SNPs explain a large proportion of the heritability for human height. Nat Genet.

[38] Pszczola M, Strabel T, Mulder HA, Calus MPL (2012). Reliability of direct genomic values for animals with different relationships within and to the reference population. J Dairy Sci.

[39] Clark SA, Hickey JM, Daetwyler HD, van der Werf JHJ (2012). The importance of information on relatives for the prediction of genomic breeding values and the implications for the makeup of reference data sets in livestock breeding schemes. Genet Sel Evol.

[40] de Haas Y, Calus MPL, Veerkamp RF, Wall E, Coffey MP, Daetwyler HD, Hayes BJ, Pryce JE (2012). Improved accuracy of genomic prediction for dry matter intake of dairy cattle from combined European and Australian data sets. J Dairy Sci.

[41] Pszczola M, Strabel T, van Arendonk JAM, Calus MPL (2012). The impact of genotyping different groups of animals on accuracy when moving from traditional to genomic selection. J Dairy Sci.

[42] Hayes BJ, Bowman PJ, Chamberlain AC, Verbyla K, Goddard ME (2009). Accuracy of genomic breeding values in multi-breed dairy cattle populations. Genet Sel Evol.

[43] Pryce JE, Gredler B, Bolormaa S, Bowman PJ, Egger-Danner C, Fuerst C, Emmerling R, Solkner J, Goddard ME, Hayes BJ (2011). Short communication: genomic selection using a multi-breed, across-country reference population. J Dairy Sci.

[44] Boneh S, Mendieta GR (1994). Variable selection in regression models using principal components. Comm Stat Theor Meth.

[45] Hawkins DM (1973). On the investigation of alternative regressions by principal component analysis. J R Stat Soc Series C (Appl Stat).

[46] Jolliffe IT (1982). A note on the use of principal components in regression. J R Stat Soc Series C (Appl Stat).

[47] Hadi AS, Ling RF (1998). Some cautionary notes on the use of principal components regression. Am Stat.

[48] Long N, Gianola D, Rosa GJM, Weigel KA (2011). Dimension reduction and variable selection for genomic selection: application to predicting milk yield in Holsteins. J Anim Breed Genet.

[49] Schölkopf B, Smola A, Müller K-R (1998). Nonlinear component analysis as a kernel eigenvalue problem. Neural Comput.

[50] Gianola D, Fernando RL, Stella A (2006). Genomic-assisted prediction of genetic value with semiparametric procedures. Genetics.

[51] Tipping ME, Bishop CM (1999). Probabilistic principal component analysis. J Roy Stat Soc B.

[52] Kim B, Kwon MJ (2008). Optimization of principal-component-analysis-applied in situ spectroscopy data using neural networks and genetic algorithms. Appl Spectrosc.

[53] Jombart T, Pontier D, Dufour AB (2009). Genetic markers in the playground of multivariate analysis. Heredity.

