# Supplementary figures and images for: A comparison of principal component regression and genomic REML for genomic prediction across populations
Source: Genet Sel Evol. 2014 Nov 5;46(1):60. doi: 10.1186/s12711-014-0060-x (PMC4220066; doi:10.1186/s12711-014-0060-x)

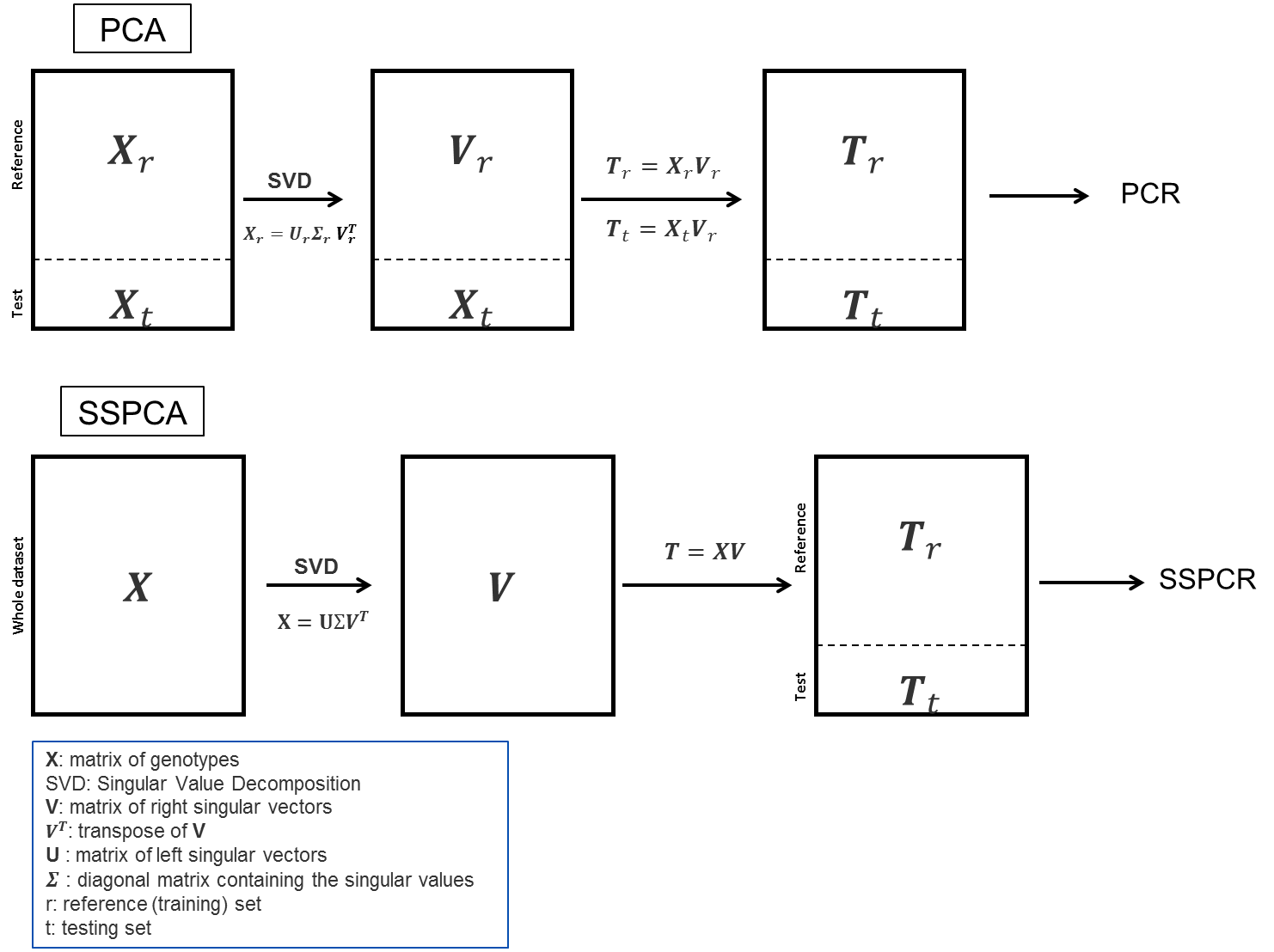

Supplement: Additional file 1: Figure S1. — Schematic overview of computation of PC in PCR and SSPCR models. PC were computed using either only genotypes of the reference data (PCR) or using genotypes of both the reference and test dataset (SSPCR). [file 12711_2014_60_MOESM1_ESM.png]
