# Supplementary material for: A comparison of principal component regression and genomic REML for genomic prediction across populations
Source: Genet Sel Evol. 2014 Nov 5;46(1):60. doi: 10.1186/s12711-014-0060-x (PMC4220066; doi:10.1186/s12711-014-0060-x)
Supplement: Additional file 2: Figure S2. — Schematic overview of stratified and 5-fold cross-validation (CV) approaches, used to select PC in the model based on minimum mean squared error of the predictions within the reference dataset. The CV was performed by splitting the reference dataset by country (stratified CV), or by splitting it randomly in a 5-fold CV. [file 12711_2014_60_MOESM2_ESM.pdf]

a)

Dataset				
Reference				Testing
UK_1	UK_2	SWE	IRL	NLD

Training			Validation
UK_2	SWE	IRL	UK_1
UK_1	SWE	IRL	UK_2
UK_2	UK_2	IRL	SWE
UK_2	UK_2	SWE	IRL

Validation	PC$_1$	PC$_2$	...	PC$_k$
UK_1	MSE$_{11}$	MSE$_{12}$	...	MSE$_{1k}$
UK_2	MSE$_{21}$	MSE$_{22}$	...	MSE$_{2k}$
SWE	MSE$_{31}$	MSE$_{32}$	...	MSE$_{3k}$
IRL	MSE$_{41}$	MSE$_{42}$	...	MSE$_{4k}$
**Average**	**(¼)MSE$_{Av,1}$**	**(¼)MSE$_{Av,2}$**	**...**	**(¼)MSE$_{Av,k}$**

By groups split CV
PCR Model selection
$argmin_{1,\ldots,k}$(MSE$_{Av}$)

PREDICTION → NLD

b)

Dataset	
Reference	Testing
UK_1 - UK_2 - SWE - IRL	NLD

5 – fold random split

1	2	3	4	5

Training				Validation
2	3	4	5	1
1	3	4	5	2
1	2	4	5	3
1	2	3	5	4
1	2	3	4	5

Validation	PC$_1$	PC$_2$	...	PC$_k$
1	MSE$_{11}$	MSE$_{12}$	...	MSE$_{1k}$
2	MSE$_{21}$	MSE$_{22}$	...	MSE$_{2k}$
3	MSE$_{31}$	MSE$_{32}$	...	MSE$_{3k}$
4	MSE$_{41}$	MSE$_{42}$	...	MSE$_{4k}$
5	MSE$_{51}$	MSE$_{52}$	...	MSE$_{5k}$
**Average**	**(1/5)MSE$_{Av,1}$**	**(1/5)MSE$_{Av,2}$**	**...**	**(1/5)MSE$_{Av,k}$**

5 – k random CV
PCR Model selection
$argmin_{1,\ldots,k}$(MSE$_{Av}$)

PREDICTION → NLD

$MSE = mean(\|y - T\hat{g}\|_2^2)$
y: observed phenotypes
T: matrix of PC scores
$\hat{g}$: estimated regression coefficients
